# Genetic Variation and Population Structure in Jamunapari Goats Using Microsatellites, Mitochondrial DNA, and Milk Protein Genes

**DOI:** 10.1100/2012/618909

**Published:** 2012-04-19

**Authors:** P. K. Rout, K. Thangraj, A. Mandal, R. Roy

**Affiliations:** ^1^Central Institute for Research on Goats, Makhdoom, Farah, Mathura 281122, India; ^2^Centre for Cellular and Molecular Biology, Uppal Road, Hyderabad 500007, India

## Abstract

Jamunapari, a dairy goat breed of India, has been gradually declining in numbers in its home tract over the years. We have analysed genetic variation and population history in Jamunapari goats based on 17 microsatellite loci, 2 milk protein loci, mitochondrial hypervariable region I (HVRI) sequencing, and three Y-chromosomal gene sequencing. We used the mitochondrial DNA (mtDNA) mismatch distribution, microsatellite data, and bottleneck tests to infer the population history and demography. The mean number of alleles per locus was 9.0 indicating that the allelic variation was high in all the loci and the mean heterozygosity was 0.769 at nuclear loci. Although the population size is smaller than 8,000 individuals, the amount of variability both in terms of allelic richness and gene diversity was high in all the microsatellite loci except ILST 005. The gene diversity and effective number of alleles at milk protein loci were higher than the 10 other Indian goat breeds that they were compared to. Mismatch analysis was carried out and the analysis revealed that the population curve was unimodal indicating the expansion of population. The genetic diversity of Y-chromosome genes was low in the present study. The observed mean M ratio in the population was above the critical significance value (Mc) and close to one indicating that it has maintained a slowly changing population size. The mode-shift test did not detect any distortion of allele frequency and the heterozygosity excess method showed that there was no significant departure from mutation-drift equilibrium detected in the population. However, the effects of genetic bottlenecks were observed in some loci due to decreased heterozygosity and lower level of M ratio. There were two observed genetic subdivisions in the population supporting the observations of farmers in different areas. This base line information on genetic diversity, bottleneck analysis, and mismatch analysis was obtained to assist the conservation decision and management of the breed.

## 1. Introduction

**Figure 1:**
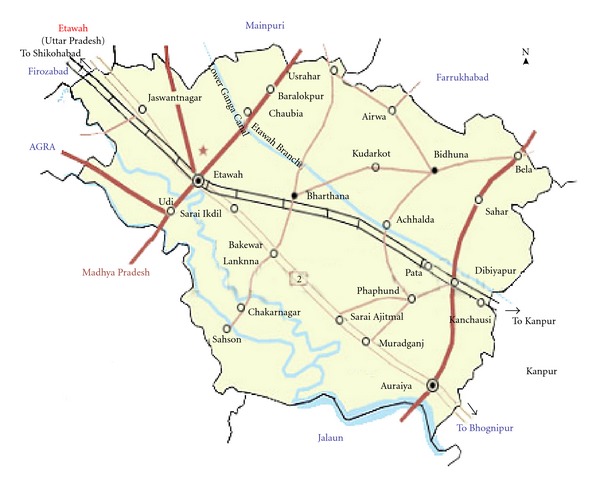
Maps of Chakarnagar (Etawah, UP) showing the home tract of Jamunapari goats.

Genetic diversity, the primary component of adaptive evolution, is essential for the long-term survival probability of a population [1–4]. Genetic diversity within domesticated species depends on several factors such as changing agricultural practices, breed replacement, and cross breeding. Genetic diversity has been analysed by using protein polymorphism, mitochondrial diversity, and microsatellite marker in both domestic and wild species [4–11]. Jamunapari goat, the majestic milk-producing goat breed of India, has suffered a reduction in numbers in its home tract [12, 13] and is considered as an endangered breed [14]. The Indian Jamunapari goat is one of the ancestors of the American Nubian and has been used in India and adjacent countries as an improver breed. The breed possesses several unique characteristics such as higher kidding rate despite its large body size. The breed inhabits isolated ravines in the Chakarnagar area of Etawah (Uttar Pradesh, India) (Figure 1), and geographical isolation has contributed towards the evolution of this unique breed. The breed is gradually declining in number due to land reclamation, decrease in grazing area, breed replacement, and the population size is less than 8,000 [12]; therefore, there is an urgent need to define strategies for conservation of this breed in its natural habitat.

In this study, we sample the Jamunapari goat population to analyse the genetic variation due to locus-specific events (selective sweep) as well as genome wide events (bottlenecks). Microsatellite markers are highly polymorphic and have been extensively used for breed diversity analysis. Mitochondrial DNA (mt DNA) and Y-chromosome region are usually sensitive to genetic drift and can be useful for detecting effects of bottlenecks in the population. Nonneutral markers are also being used to analyse population diversity, and milk protein gene has been used as the region is directly involved for the survival of the individual and under strong selective pressure. By integrating data from multiple markers, we provide the possible factors affecting the genetic consequence of population reduction in this breed.

## 2. Materials and Methods

Fifty blood samples were collected in 10 villages in which the breed has a major concentration. Samples were collected from the individuals exhibiting typical breed characteristics such as white colour, Roman nose, and pendulous ear (farmers are not selecting for these traits) and at least two samples were collected from each village. An effort was made to collect samples from unrelated individuals based on information provided by farmers. The breeding buck is available with one or two farmers in every village, and some farmers also maintain breeding bucks during breeding season, disposing of them after the breeding season. Blood samples were collected from each animal using EDTA vacutainer and stored at –20°C till further use.

Microsatellite analysis was carried out to test for signatures of recent population bottlenecks in Jamunapari goats. This analysis was carried out on 49 DNA samples with 17 microsatellite markers (Table 1) as reported by Rout et al. [11]. For these 17 loci, genetic variation was quantified using measures of the total number of alleles, number of polymorphic loci, observed and expected heterozygosity per locus, and allelic richness using GENEPOP (Version 3.4; [15]), FSTAT2.93 [16], and AGA_rst_ [17]. Heterozygosity was measured as the mean observed heterozygosity (Ho) and the mean expected heterozygosity (H_E_) based on Hardy-Weinberg assumptions. We tested genotypic linkage disequilibrium between all pairs of loci in each population with GENEPOP (Version 3.4; [15]) based on Markov chain method with 10,000 iterations and 100 batches. We also used FSTAT software to assess 95% confidence intervals of Weir and Cockerham's f, which measures deviation from the Hardy-Weinberg equilibrium (HWE) for populations and corresponds to Wright's within-population inbreeding coefficient F_IS_.

Milk protein genes, which are expected to be nonneutral markers, were also used to analyse the population variability. Two milk protein genes, namely, *β-LG* gene and *CSN1S1* (*α*s_1_-casein) were analysed using PCR-RFLP to observe genetic variability in 35 individuals. The *α*s_1_-casein (*CSN1S1*) gene produced an amplified fragment of 223 bp which was digested with the Xmn*I* restriction enzyme. The *β*-LG gene produced an amplified product of 426 bp, and RFLP analysis was carried out with the Sac*II* restriction enzyme. The PCR-RFLP analysis was carried out as described by Kumar et al. [18, 19], and the data were analysed separately for mean number of alleles, expected heterozygosity and Hardy-Weinberg equilibrium (HWE) using POPGENE software [20].

mtDNA HVRI sequencing was carried out as described by Joshi et al. [10]. Four hundred and fifty-seven base pairs from the mtDNA HVRI regions of 50 individuals were aligned using CLUSTAL X. We used mismatch distribution [21] to analyse the population expansion as implemented in ARLEQUIN 3.1 [22]. Fu's F value was calculated from mtDNA haplotypes to test for deviations from neutral equilibrium condition [23]. The qualitative and quantitative aspect of the population's genetic history may be uncovered by the analysis of frequency distributions of pairwise sequence mismatches. Mismatch analysis (the distribution of all pair-wise nucleotide differences between sequences) was carried out to test the deviation of the observed data from neutral predictions expected in constant-sized populations.

Genetic divergence was analysed by selecting three primers from ovine male-specific region (AMLEY, SRY, and ZFY gene) [24]. PCR was carried out in a 50 *μ*L reaction volume containing 100 ng of DNA, 20 pM of each primer, 200 *μ*M of dNTP, 2 mM Mgcl_2_, and %U of Taq DNA polymerase (New India Biolab, MA, USA). The samples were subjected to sequencing after purifying the PCR product by gene elute PCR clean up kit. Individual PCR amplified products were subjected to sequencing in 12 samples. PCR products were sequenced on both the strands directly using 50 ng (2.0 *μ*L) of PCR product and 4 pM (1.0 *μ*L) of primer, 4 *μ*L of Big Dye Terminator ready reaction kit (Perkin Elmer, Foster City, USA), and 3.0 *μ*L of double distilled water to adjust the volume to 10.0 *μ*L. Cycle sequencing was carried out in a Gene Amp 9600 thermal cycler (Perkin Elmer) employing the PCR conditions. Extended products were purified by alcohol precipitation followed by washing with 70% alcohol. Purified samples were dissolved in 10 *μ*L of 50% Hi-Di formamide and analysed in an ABI 3700 automated DNA Analyzer (Perkin Elmer, USA). Nucleotide diversity, expected heterozygosity, Tajima's D, and Fu's Fs values were estimated in ARLEQUIN 3.1 [22].

 Genetic bottleneck was detected using microsatellite data by three approaches, heterozygote excess, mode-shift, and M ratio test. We first used the M ratio (the mean ratio of the number of alleles to total range in allele size) [25] as implemented in AGA_rst_ [17], because of its consistent performance in identifying populations with known bottlenecks. M ratio calculates the changes that occur after a bottleneck in the distribution of allele sizes relative to the number of alleles in a population. It has been established that an M ratio less than 0.71 signifies a bottleneck [25].

The BOTTLENECK programme [26] was used as an alternative measure of genetic bottlenecks to test for excess gene diversity relative to that expected under mutation-drift equilibrium. The heterozygosity excess method exploits the fact that allele diversity is reduced faster than heterozygosity during a bottleneck, because rare alleles are lost rapidly and have little effect on heterozygosity, thus producing a transient excess in heterozygosity relative to that expected in a population of constant size with the same number of alleles [26, 27]. To determine the population “genetic reduction signatures” characteristic of recent reductions in effective population size (Ne), the Wilcoxon's heterozygosity excess test [26] and the allele frequency distribution mode shift analysis [28] were performed using BOTTLENECK [26]. The heterozygosity excess method was used to analyse the population, and the data for the heterozygosity excess test were examined under the two-phased model (TPM; 95% stepwise mutation model with 5% multistep mutations and a variance among multiple steps of 12), which is considered best for microsatellite data [26, 29]. We also analysed the allele frequency distribution for gaps. A qualitative descriptor of allele frequency distribution (the mode-shift indicator), which is reported to discriminate between bottlenecked and stable population [28], was obtained using the programme BOTTLENECK.

We used an individual-based clustering approach (STRUCTURE 2.1, [30]) to determine the most likely number of genetic clusters (*k*) in the Jamunapari populations. STRUCTURE software sorts individual genotypes into clusters that maximize the fit of the data to theoretical expectation. Based on preliminary analyses, we evaluated the likelihood of *k* = 2 and *k* = 3, with 5 runs performed for each *k*, and a burn-in length of 500,000 and 100,000 MCMC replicates for each run. We assumed an admixture model and correlated allele frequencies among populations [30].

## 3. Results and Discussion

The markers with their chromosome number, number of alleles identified, and allele size range have been described in Table 1. Among the polymorphic markers, BM4621 and IDVGA7 showed highest number of alleles (15) at each locus. The number of alleles per microsatellite marker was above 6 for all markers except for ILSTS005 and Oar HH62. The total number of alleles was 153 over the 17 loci. The allelic richness ranged from 3.00 to 15.00 across the microsatellite markers (Table 1) and the mean number of alleles per locus was 9.0. Allelic richness was identical to allele frequency implying that there was no bias based on sample size. The average gene diversity ranged from 0.489 to 0.866 over the loci. The mean expected and observed heterozygosity was 0.769 and 0.386 (Table 1). All the loci showed higher gene diversity than ILSTS005 in the analysed samples. The high mean number of alleles per locus and expected heterozygosities indicated that the overall gene diversity was high in the population. Heterozygosity and allele number are aligning with high diversity score in the population. Takezaki and Nei (1996) suggested that microsatellite loci can be included diversity analysis having heterozygosity from 0.3 to 0.8 in the population. Two loci departed significantly from the Hardy-Weinberg equilibrium (HWE). In the analysed samples, 18 microsatellite locus pairs demonstrated linkage disequilibrium (LD) with *P* value <0.05. The LD was significant in 13.21% of the locus pair combinations in the population. The overall excess of homozygosity for the population as a whole varied from 0.089 to 0.785 over the loci and the average was 0.500. The high levels of allelic diversity are coupled with very high F_is_ indicating that the population is experiencing high levels of nonrandom mating in the breeding tract but simultaneously maintaining allelic diversity over the entire range of the breed. Gour et al. [31] also observed high inbreeding in Jamunapari goats; further, high estimates of inbreeding have been reported for Asian goat populations by Barker et al. [8]. 

Genetic variation at *CSN1S1* and *β*-LG was 0.395 and 0.107. The effective number of alleles was 1.653 and 1.20 at *CSN1S1* and *β*-LG loci, respectively. The *β*-LG locus showed significant departure from HW equilibrium. The gene diversity and effective number of alleles at milk protein loci were higher than for 10 other Indian goat breeds, supporting the fact that the breed maintains higher genetic variability [18, 19].

The population was examined for allele frequency distribution for gaps, and M ratios are presented in Table 2. The M ratios ranged from 0.364 to 1.00 with an average of 0.815, which was significantly higher than the critical value. The M ratio was less than 0.71, diagnostic value of genetic bottlenecks, in the case of ILSTS005, INRABERN192, and BM143. The observed M ratio for all other markers in the population was very high and close to one indicating that it is a very slowly changing population (at least not showing the sign of bottleneck).

**Figure 2:**
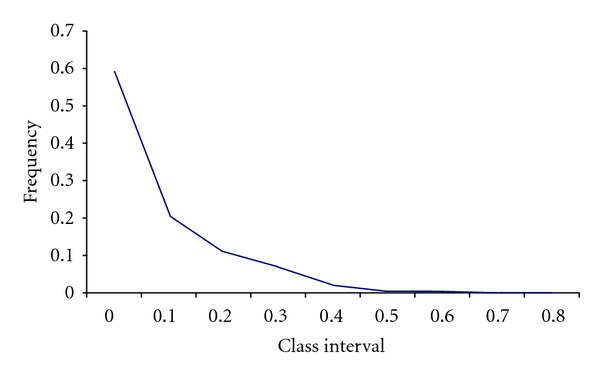
L-shaped mode shift graph showing the absence of bottleneck in Jamunapari goats.

Bottleneck detection in Jamunapari goat was presented in Table 2. The mode shift test did not detect any distortion of allele frequency and showed a normal “L” shaped distribution which is a typical property of a population in equilibrium (Figure 2). The heterozygosity excess method was carried out to analyse historical bottlenecks. Out of 17 loci, 7 loci showed heterozygosity excess (Wilcoxon signed rank test, *P* = 0.8487, one tail for heterozygosity excess), and there was no significant departure from mutation-drift equilibrium detected in the population. Under the two-phase mutation model equilibrium, each individual has a roughly equal chance of having heterozygote deficiency or excess. The analysis indicated that the population has not suffered any bottleneck recently and was a constant size population. The present study agrees well with the observation of Gour et al. [31].

The sequence analysis of the 457 bp mitochondrial HVRI region identified 34 mtDNA haplotypes, and the overall haplotype diversity was 0.984. The mtDNA variation detected relatively high number of haplotypes from a total 50 individuals, and haplotype diversity was quite high. Fu's Fs value is based on the probability of recovering a number of haplotypes greater than or equal to the observed number in a sample drawn from a stationery population with the same mean number of pairwise differences as the observed sample. Fu's Fs value was −15.53. The significant negative Fs values indicated the large and sudden expansion in the population at geographical locations. The population showed a significant negative Fs value indicating excess of rare mutations, a pattern commonly attributed to a normal growing population.

**Figure 3:**
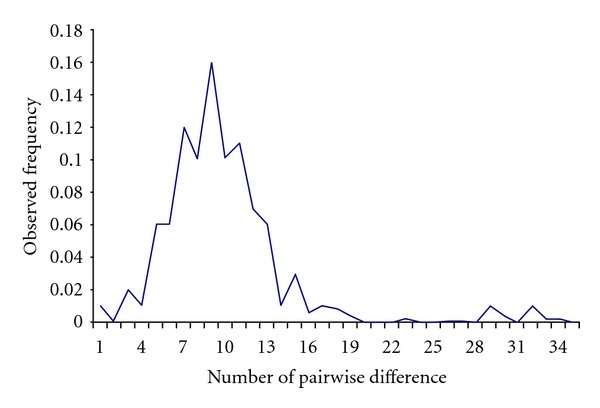
Mismatch distribution in Jamunapari goats analysed for mt-DNA control region.

Mismatch distribution analysis revealed the genetic signature of population expansion of Jamunapari goat. The tau value with 95% confidence interval was 8.20 (5.22–10.68), and the Sum of Square Difference (SSD) value was 0.0048. The SSD value showed large population expansion in Jamunapari goats.  Figure 3 depicts the mismatch distribution of Jamunapari goat. The shape of the distribution of number of observed differences between pairs of DNA sequences showed almost unimodal curve for the Jamunapari goat. The curve showed very small second mode towards the end indicating that some minor population expansion at some stage might have occurred in various geographical areas. Mismatch distribution has been extensively used to estimate the demographic parameters of past population expansion or contraction as it leaves a recognizable signature in the pattern of molecular diversity [21, 32, 33]. The unimodal distribution of pairwise differences in the breed (Figure 3) and Fu's Fs value indicated a sudden demographic population expansion and origin of the breed from a limited number of founder populations. Migration had an effect on the shape of curve, and intermediate migration rate would have led to a multimodal curve [34]. However, the factors like inbreeding and admixture with other population could affect the shape of mismatch distribution. 

The amplified product for amelogenin gene (AMELY), SRY gene, and ZFY gene was 733 bp, 632 bp, and 584 bp, respectively. No diversity was observed in SRY and AMELY genes. Nucleotide diversity and expected heterozygosity were 0.135 ± 0.089 and 0.1384, respectively, for ZFY gene. Similarly Tajima's D value and Fu's Fs value were zero for SRY and AMELY gene. Tajima's D value and Fu's Fs value were 0.6797 and 2.539, respectively and non-significant for ZFY gene. Transition and transversion ratio was 47 : 90 for ZFY gene. ZFY gene showed low gene diversity and positive Tajima's D value and Fu's Fs value. Y-linked nucleotide diversity was found low in human, wolf, cattle, reindeer and Lynx indicating that reduced levels of Y-chromosome polymorphism may be a generalized feature of mammalian genome [35]. Y chromosome variability is expected to be lowest as compared to autosomes and X-chromosome. The major factors explain that the low levels of Y chromosome variability are selection, mating system, or migration patterns, or other mechanisms lowering male effective population size. The low levels of Y chromosome variability that we found in goat could be attributed to a strong sex bias in breeding.

The proportion of membership of individuals in the Jamunapari population placed 12.6% of population into one cluster group in both STRUCTURE analyses (*k* = 2 or 3). The cluster defined by STRUCTURE showed clear membership of individuals in two clusters indicating a genetic subdivision within the breed. There was a difference between expected and observed heterozygosity supporting the genetic subdivision within the breed. It has been also observed during survey that there are two strains of Jamunapari locally known as “Kathey” and “Kandhan”. Kathey-type goats have long thick broad and less folded ears with thick and flat neck. Kandhan-type goats have long, soft, and folded ears, and the neck is thin and cylindrical. Kandhan is restricted to river banks of Chambal and Kathey is distributed throughout the breeding tract. (CIRG Report).

The average M ratio was large and above the critical significance value (Mc) in the population suggesting that it has not suffered severe or long-lasting genetic bottlenecks [25, 36]. Heterozygosity excess test and the mode shift indicators demonstrated that there was no sign of recent reductions in effective population size (Ne) in the population. Moreover, the milk protein loci also exhibited higher gene diversity at both the loci as compared to other Indian goat breeds [18, 19]. Moreover, the mtDNA analysis supported population expansion model as evidenced from Fu's Fs value, unimodal distribution of pairwise differences (mismatch curve). Genetic variation is often reduced due to demographic reduction in population and can also show other signs of population bottlenecks. However, this breed showed retention of diversity in the face of population reduction. A similar type of trend had been observed in salmon population [37] and turtle [38]. Genetic responses to bottlenecks depend on life history and the life-span of the species, the severity of the demographic decline, level of present gene flow, and nature of demographic rebound [25, 39]. Several factors might be affecting the population leading to maintenance of dramatic genetic variation despite severe demographic declines. The variation may be due to exchange of animals between different areas and selection criteria of farmers to use the individuals as parents for the next generation. The dispersal of local populations between farmers and selling to outside agencies may have served to maintain genetic variability in the population. The breeding buck is available with one or two farmers in every village and all the farmers mate their goats with the available bucks showing the nonrandom mating in the breeding tract but maintaining allelic diversity over the entire range of the breed. Again the existence of genetic subdivision supports the existing gene diversity in the population. Historically there was no evidence of a major earthquake or climate change in the area. Theoretically, the impact of even severe bottlenecks can be small if the bottleneck is followed by the rapid flush of growth in which most genetic variability is maintained [39]. Again, the population growth is evident but also animals are supplied to outside the home tract as a medium of remunerative income to farmers. Additionally, the overlapping generations in domestic goats buffer them from long-term losses of genetic variability in comparison to species with discrete generations. More importantly, the high reproductive rate of this large goat breed slows down the losses due to genetic drift from the base population. Small populations are generally considered to be susceptible to a number of genetic problems like low level of variability, inbreeding depression, and the ability to overcome disease agents; however, the population did not exhibit any such effect over the years in the adopted villages [12, 13]. The population has not shown any noticeable physiological sign of inbreeding depression as there was no reduction in fecundity (kidding rate); and the mortality in the breeding tract over the year was also low (<7.5%, [12]). It was observed that the rate of decline in genetic diversity was relatively slow in the population in its natural breeding tract; however, the low genetic diversity in Y chromosomal genes, as well as the effects of genetic bottlenecks in some loci due to decreased heterozygosity and lower level of M ratio, supports population reduction in breeding tract. Conservation will be much more difficult when the population becomes genetically impoverished and is effective and easy to implement when the populations are genetically stable. Therefore, it is necessary to initiate necessary steps to conserve the breed for future use.

## Figures and Tables

**Table 1:** Microsatellite markers and chromosomal location, total number of alleles and genetic diversity in the Jamunapari goats.

Markers	Chromosome number	Observed number of alleles	Allele size range (bp)	Gene diversity	Allelic richness	F_IS_
BM4621	6	15	106–140	0.862	15.00	0.652
NRAMP	2	10	224–248	0.807	10.00	0.554
OarAE101	6	8	92–108	0.809	8.00	0.555
IDVGA7	25	15	210–240	0.890	15.00	0.573
ILSTS005	10	3	178–188	0.497	3.00	0.235
BM6526	27	9	154–178	0.801	9.00	0.500
ETH225	14	6	140–152	0.703	6.00	0.089
OarHH56	23	9	152–168	0.818	9.00	0.560
INRABERN192	7	10	178–208	0.823	10.00	0.417
OarFCB48	17	8	150–164	0.831	8.00	0.351
OarHH62	20	5	108–118	0.719	5.00	0.499
TGLA40		10	174–198	0.782	10.00	0.540
BM143	6	8	96–118	0.741	8.00	0.514
SRCRSP 5	21	8	160–178	0.794	8.00	0.748
SRCRSP6	19	10	138–158	0.680	10.00	0.530
SRCRSP9		11	120–144	0.877	11.00	0.247
SRCRSP10	8	9	260–276	0.836	9.00	0.785

**Table 2:** Bottleneck detection in the Jamunapari goats.

Marker	Heq*	SD	*(He-Heq)/SD	He excess	M ratio*
BM4621	0.894	.021	−1.825	+	0.833
NRAMP	0.834	.036	−0.886	+	0.692
OarAE101	0.787	.050	0.337	−	0.889
IDVGA7	0.894	.021	−0.436	+	0.938
ILSTS005	0.441	.151	0.364	−	0.364
BM6526	0.808	.048	−0.244	+	0.692
ETH225	0.714	.074	−0.159	+	0.857
OarHH56	0.813	.043	−0.007	+	0.857
INRABERN192	0.833	.038	−0.359	+	0.625
OarFCB48	0.784	.053	0.845	−	1.00
OarHH62	0.653	.093	0.672	−	0.833
TGLA40	0.831	.041	−1.275	+	0.769
BM143	0.786	.053	−0.934	+	0.667
SRCRSP5	0.786	.052	0.051	−	0.800
SRCRSP6	0.830	.037	−4.182	+	0.909
SRCRSP9	0.849	.034	0.763	−	0.846
SRCRSP10	0.813	.041	0.393	−	1.000

*Heq is the heterozygosity expected at equilibrium obtained through coalescent simulation under the “two-phase mutation model”. (He-Heq)/SD: the standardized difference for each locus, M ratio: the number of allele/(range in allele size + 1).
